# SARS-CoV-2 Point Mutation and Deletion Spectra and Their Association with Different Disease Outcomes

**DOI:** 10.1128/spectrum.00221-22

**Published:** 2022-03-29

**Authors:** Brenda Martínez-González, María Eugenia Soria, Lucía Vázquez-Sirvent, Cristina Ferrer-Orta, Rebeca Lobo-Vega, Pablo Mínguez, Lorena de la Fuente, Carlos Llorens, Beatriz Soriano, Ricardo Ramos, Marta Cortón, Rosario López-Rodríguez, Carlos García-Crespo, Isabel Gallego, Ana Isabel de Ávila, Jordi Gómez, Luis Enjuanes, Llanos Salar-Vidal, Jaime Esteban, Ricardo Fernandez-Roblas, Ignacio Gadea, Carmen Ayuso, Javier Ruíz-Hornillos, Nuria Verdaguer, Esteban Domingo, Celia Perales

**Affiliations:** a Department of Clinical Microbiology, Instituto de Investigación Sanitaria-Fundación Jiménez Díaz University Hospital, Universidad Autónoma de Madrid (IIS-FJD, UAM), Madrid, Spain; b Centro de Biología Molecular “Severo Ochoa” (CSIC-UAM), Consejo Superior de Investigaciones Científicas (CSIC), Madrid, Spain; c Structural Biology Department, Institut de Biología Molecular de Barcelona CSIC, Barcelona, Spain; d Department of Genetics & Genomics, Instituto de Investigación Sanitaria-Fundación Jiménez Díaz University Hospital, Universidad Autónoma de Madrid (IIS-FJD, UAM), Madrid, Spain; e Centre for Biomedical Network Research on Rare Diseases (CIBERER), Instituto de Salud Carlos III, Madrid, Spain; f Bioinformatics Unit, Instituto de Investigación Sanitaria-Fundación Jiménez Díaz University Hospital, Universidad Autónoma de Madrid (IIS-FJD, UAM), Madrid, Spain; g Biotechvana, “Scientific Park”, Universidad de Valencia, Valencia, Spain; h Unidad de Genómica, “Scientific Park of Madrid”, Campus de Cantoblanco, Madrid, Spain; i Centro de Investigación Biomédica en Red de Enfermedades Hepáticas y Digestivas (CIBERehd), Instituto de Salud Carlos III, Madrid, Spain; j Instituto de Parasitología y Biomedicina ‘López-Neyra’ (CSIC), Parque Tecnológico Ciencias de la Salud, Granada, Spain; k Department of Molecular and Cell Biology, Centro Nacional de Biotecnología (CNB-CSIC), Consejo Superior de Investigaciones Científicas (CSIC), Madrid, Spain; l Allergy Unit, Hospital Infanta Elena, Valdemoro, Madrid, Spain; m Instituto de Investigación Sanitaria-Fundación Jiménez Díaz University Hospital, Universidad Autónoma de Madrid (IIS-FJD, UAM), Madrid, Spain; n Faculty of Medicine, Universidad Francisco de Vitoria, Madrid, Spain; Fundacio irsiCaixa

**Keywords:** COVID-19 severity, mutant spectrum, diversity index, mutation, deletion, nsp12 (polymerase), spike, ultradeep sequencing

## Abstract

Mutant spectra of RNA viruses are important to understand viral pathogenesis and response to selective pressures. There is a need to characterize the complexity of mutant spectra in coronaviruses sampled from infected patients. In particular, the possible relationship between SARS-CoV-2 mutant spectrum complexity and disease associations has not been established. In the present study, we report an ultradeep sequencing (UDS) analysis of the mutant spectrum of amplicons from the nsp12 (polymerase)- and spike (S)-coding regions of 30 nasopharyngeal isolates (diagnostic samples) of SARS-CoV-2 of the first COVID-19 pandemic wave (Madrid, Spain, April 2020) classified according to the severity of ensuing COVID-19. Low-frequency mutations and deletions, counted relative to the consensus sequence of the corresponding isolate, were overwhelmingly abundant. We show that the average number of different point mutations, mutations per haplotype, and several diversity indices was significantly higher in SARS-CoV-2 isolated from patients who developed mild disease than in those associated with moderate or severe disease (exitus). No such bias was observed with RNA deletions. Location of amino acid substitutions in the three-dimensional structures of nsp12 (polymerase) and S suggest significant structural or functional effects. Thus, patients who develop mild symptoms may be a richer source of genetic variants of SARS-CoV-2 than patients with moderate or severe COVID-19.

**IMPORTANCE** The study shows that mutant spectra of SARS-CoV-2 from diagnostic samples differ in point mutation abundance and complexity and that significantly larger values were observed in virus from patients who developed mild COVID-19 symptoms. Mutant spectrum complexity is not a uniform trait among isolates. The nature and location of low-frequency amino acid substitutions present in mutant spectra anticipate great potential for phenotypic diversification of SARS-CoV-2.

## INTRODUCTION

Betacoronavirus SARS-CoV-2 emerged in the human population in 2019, and it is the causal agent of the new pandemic disease COVID-19 (1), with a death toll that is increasing at the time of this writing (https://covid19.who.int/). Genetic variations in SARS-CoV-2 genomes (annotated in the GISAID [https://www.gisaid.org/], PubMed [https://www.ncbi.nlm.nih.gov/pmc/], and ENA data banks [https://www.ebi.ac.uk/ena/browser/home], among others) affect nonstructural and structural protein-coding regions. Despite the short history of SARS-CoV-2 circulation, newly arising variants exhibiting different mutational patterns are being identified regularly. A distinction has been made between variants of interest (VOI), due to features with potential impact (such as transmissibility), and variants of concern (VOC), due to definite evidence of enhanced transmissibility (https://www.who.int/en/activities/tracking-SARS-CoV-2-variants/). New SARS-CoV-2 variants are likely to become prominent as COVID-19 continues, despite natural or vaccine-induced immunity (2–5). Likewise, the generation of viral escape mutants is a major concern as a potential limitation of immune and antiviral agent efficacy for SARS-CoV-2 (6–10), as it has been established for other RNA viruses.

The first step in the diversification of viruses during their epidemic spread is the generation of variants within each infected host. This pattern of intrahost evolution results in the formation of mutant spectra that constitute reservoirs of genetic and phenotypic virus variants in the infected host (11, 12). Studies with several RNA viruses have shown that viral intramutant spectrum complexity, estimated by the average number of mutations per genome, expressed by a series of diversity indices (Shannon entropy, maximum mutation frequency, Gini Simpson, nucleotide diversity, number of polymorphic sites, and number of haplotypes [13, 14]), may have an impact on viral tropism, viral persistence, disease progression, and response to antiviral interventions (several cases have been described or reviewed in references 11 and 15–22). Evidence of quasispecies dynamics has been reported for SARS-CoV-2 (23–29), as well as for other coronaviruses (30–34). However, it is unclear how mutant spectrum complexity parameters of this emerging pathogen vary among different viral isolates and whether previously observed effects of mutant spectrum composition on RNA virus behavior apply also to SARS-CoV-2, particularly its connection with disease severity.

Two recent studies indicated that mutant spectrum complexity in SARS-CoV-2 from patients who developed severe disease is higher than that from patients with mild disease, analyzing either the spike (S)-coding regions (35) or the entire genome with limited mutant spectrum resolution (36). In the present study, we have examined mutant spectra of the nsp12 (polymerase)- and S-coding regions of SARS-CoV-2 present in 30 nasopharyngeal swab samples taken at the time of diagnosis of patients progressing toward disparate disease outcomes. Applying a 0.5% cutoff value for point mutation and deletion detection, using SeekDeep as bioinformatics platform, we found that virus from patients who developed mild disease exhibited a significantly higher mutant spectrum complexity than virus from patients who developed moderate or severe disease (exitus). The difference occurred in both the nsp12 (polymerase)- and S-coding regions. In contrast, no significant differences in the spectrum of minority deletions were observed among virus from the three patient categories (mild, moderate, or severe disease). Some amino acid substitutions found at low frequency in mutant spectra, including substitutions with low statistical acceptability and with potential functional effects, are nevertheless present in SARS-CoV-2 isolates recorded in data banks.

## RESULTS

### SARS-CoV-2 mutant spectra from patients progressing toward different COVID-19 severity.

We previously classified 448 patients (Fundación Jiménez Díaz [FJD] cohort, Madrid, Spain, April 2020) according to the COVID-19 severity into three categories, mild, moderate, and severe COVID-19, based on a number of demographic and clinical parameters, and we found a positive association between viral load in nasopharyngeal swabs and disease severity (37). For the present study, we have chosen 30 of the nasopharyngeal samples based on three criteria: (i) the COVID-19 category, including 10 patients who developed mild symptoms, 10 patients who developed moderate disease, and 10 patients who progressed to severe disease and exitus, (ii) patients whose diagnostics (real-time PCR RNA samples) displayed similar cycle threshold [*C_T_*] values (average *C_T_* of 25.37 ± 3.9 for mild, *C_T_* of 21.81 ± 2.4 for moderate, and *C_T_* of 20.38 ± 2.9 for exitus patients), and (iii) similar time interval between symptom onset and swab collection (average 5.78 ± 4.2 days for mild, 4.89 ± 3.1 days for moderate, and 4.5 ± 2.6 days for exitus patients). When present, comorbidities were equally represented among the different COVID-19 severities (Table S1 in https://saco.csic.es/index.php/s/8GH5aJgritCjEx5).

**FIG 1:**
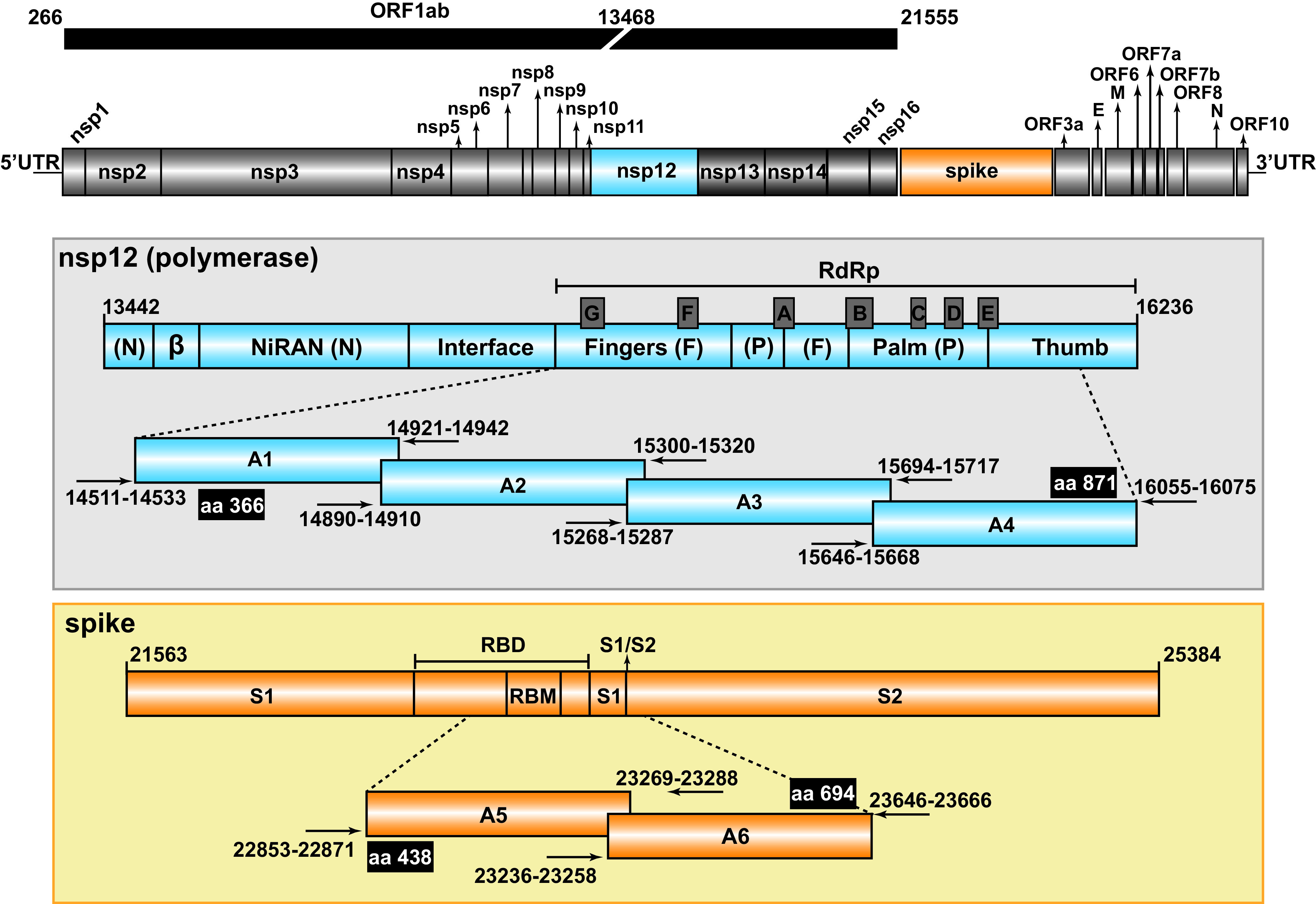
Representation of the SARS-CoV-2 genome, encoded proteins, and amplicons analyzed by UDS. The region corresponding to the ORF1ab of the virus is shown at the top. In the two boxes, the nsp12 (blue) and spike (orange) have been expanded, with the first and last nucleotide number given at the beginning and the end of the bars, respectively (genome numbering is according to the reference genome NCBI accession number: NC_045512.2). Relevant protein domains are indicated, including motifs A to G depicted as protruding gray boxes in nsp12 (polymerase) and the receptor-binding motif (RBM) and the S1/S2 cleavage site in S. The amplicons (A1 to A4 for the nsp12 [polymerase] and A5, A6, for S) are shown flanked by horizontal arrows that mark the position of the oligonucleotide primers used for amplification (oligonucleotide sequences are given in Table S5 in https://saco.csic.es/index.php/s/8GH5aJgritCjEx5). Flanking black boxes indicate the amino acids (aa) of nsp12 (polymerase) and S covered by the amplicons.

To set up ultradeep sequencing (UDS) analyses of SARS-CoV-2 obtained from nasopharyngeal swabs, we have adapted experimental protocols previously used for hepatitis C virus (HCV) quasispecies characterization (38–41) and applied the SeekDeep pipeline (42) to the analysis of minority point mutations and deletions in SARS-CoV-2 mutant spectra (described in Materials and Methods). RNA from nasopharyngeal swabs was extracted, amplified, and subjected to UDS using MiSeq platform (Illumina). Four amplicons (A1 to A4) covering nucleotides 14,534 to 16,054 of the nsp12 (polymerase)-coding region and two amplicons (A5 and A6) covering nucleotides 22,872 to 23,645 of the S-coding region were analyzed (Fig. 1). The total number of clean reads was 19,592,197, corresponding to 653,073 (range 316,710 to 910,727) reads per patient, which yielded an average of 110,689 (range 38,865 to 215,662) clean reads per amplicon, with a 0.5% cutoff frequency for point mutations and deletions (Fig. S1 in https://saco.csic.es/index.php/s/8GH5aJgritCjEx5).

**FIG 2:**
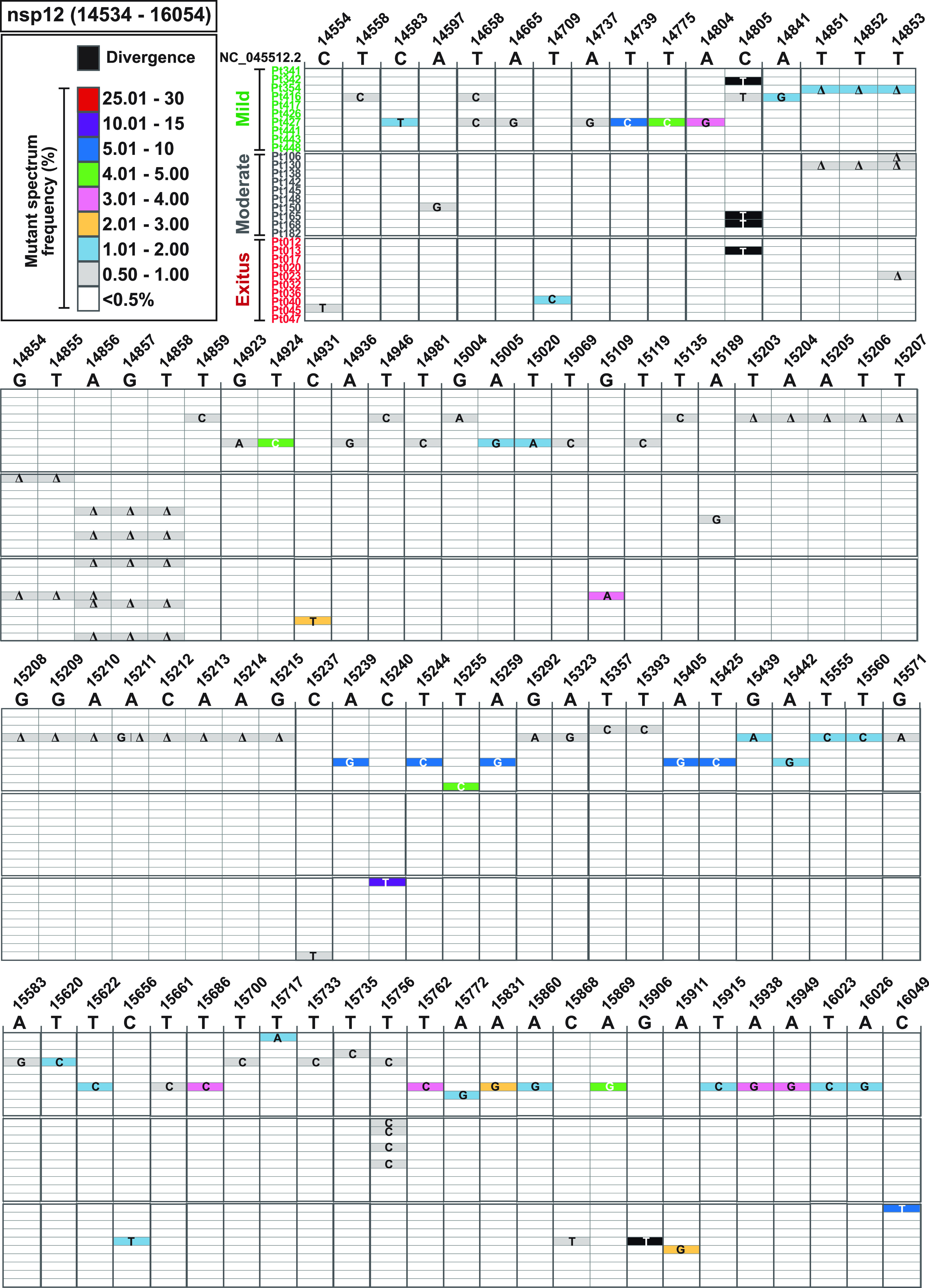

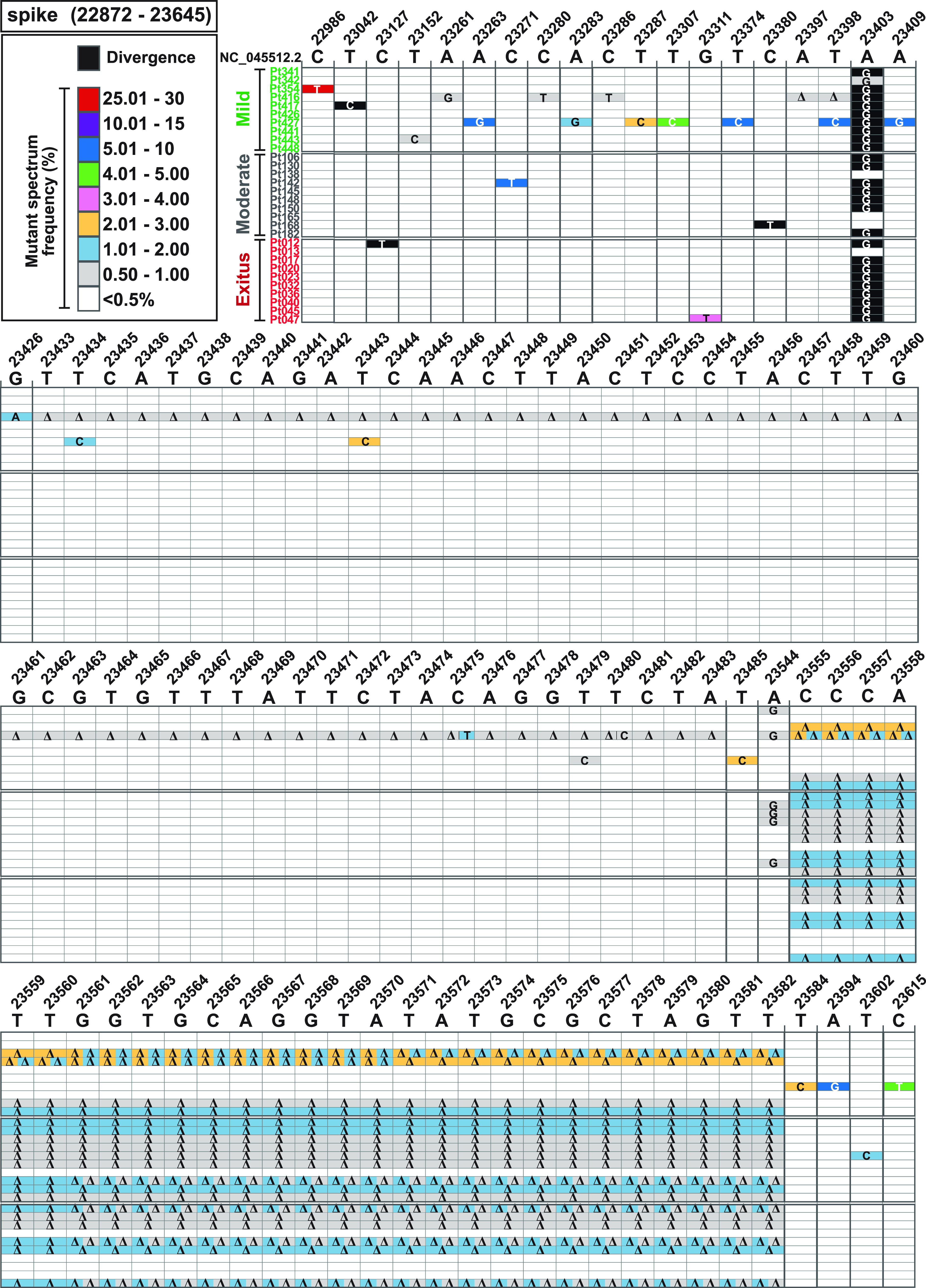
Heat map of point mutation and deletion frequencies in mutant spectra of SARS-CoV-2 from individual patients. Data are presented in two blocks, one for the nsp12 (polymerase)-coding region (genomic residues 14,534 to 16,054) and another for the S-coding region (genomic residues 22,872 to 23,645). Only positions with a mutation or those affected by a deletion are represented. Each row corresponds to a patient, and patients have been divided into those with mild, moderate, and exitus disease outcomes (color coded and with the patient identification code written at the left of each row). The patients’ clinical status and demographic data are described in Table S1 in https://saco.csic.es/index.php/s/8GH5aJgritCjEx5. Mutations and deletions have been identified relative to NCBI reference sequence NC_045512.2. Each mutation and deletion (Δ) with a frequency above the cutoff level (0.5%) is indicated, and its frequency within the mutant spectrum retrieved from each patient has been visualized with a color code displayed in the heading boxes (top left of the two blocs). Procedures are detailed in Materials and Methods.

To provide a general picture of SARS-CoV-2 divergence and mutant spectrum heterogeneity, we constructed a heat map representing the frequency of each variation in the nsp12 (polymerase)- and S-coding regions (point mutations and deletions; no insertions were detected), relative to the genomic sequence of a Wuhan isolate (identified as NCBI reference sequence number NC_045512.2), and divided the samples according to different COVID-19 severities (Fig. 2 and Table S2 in https://saco.csic.es/index.php/s/8GH5aJgritCjEx5). Considering all patients analyzed, the number of positions that included a variation (either a point mutation or a deletion) was 2-fold higher in the S-coding region (105 positions with a genomic modification out of 774 positions analyzed) than in the nsp12 (polymerase)-coding region (91 positions modified out of 1,521 positions analyzed). In addition to minority mutations in each mutant spectrum, a total of six different dominant mutations relative to the reference sequence (those with frequencies between 90% and 100%) were also present; they are identified as “Divergence” in Fig. 2. This class of mutations has been excluded for the quantification of mutations and complexity indices in a mutant spectrum. Ninety-four percent of mutations were found at frequencies that ranged between 0.5% and 30% within its mutant spectrum, whereas only 6% corresponded to divergence mutations (*P < *0.001; proportion test). Interestingly, 62 out of 97 point mutations (64%) within the mutant spectra were detected at frequencies below 2% (Fig. 2).

**FIG 3:**
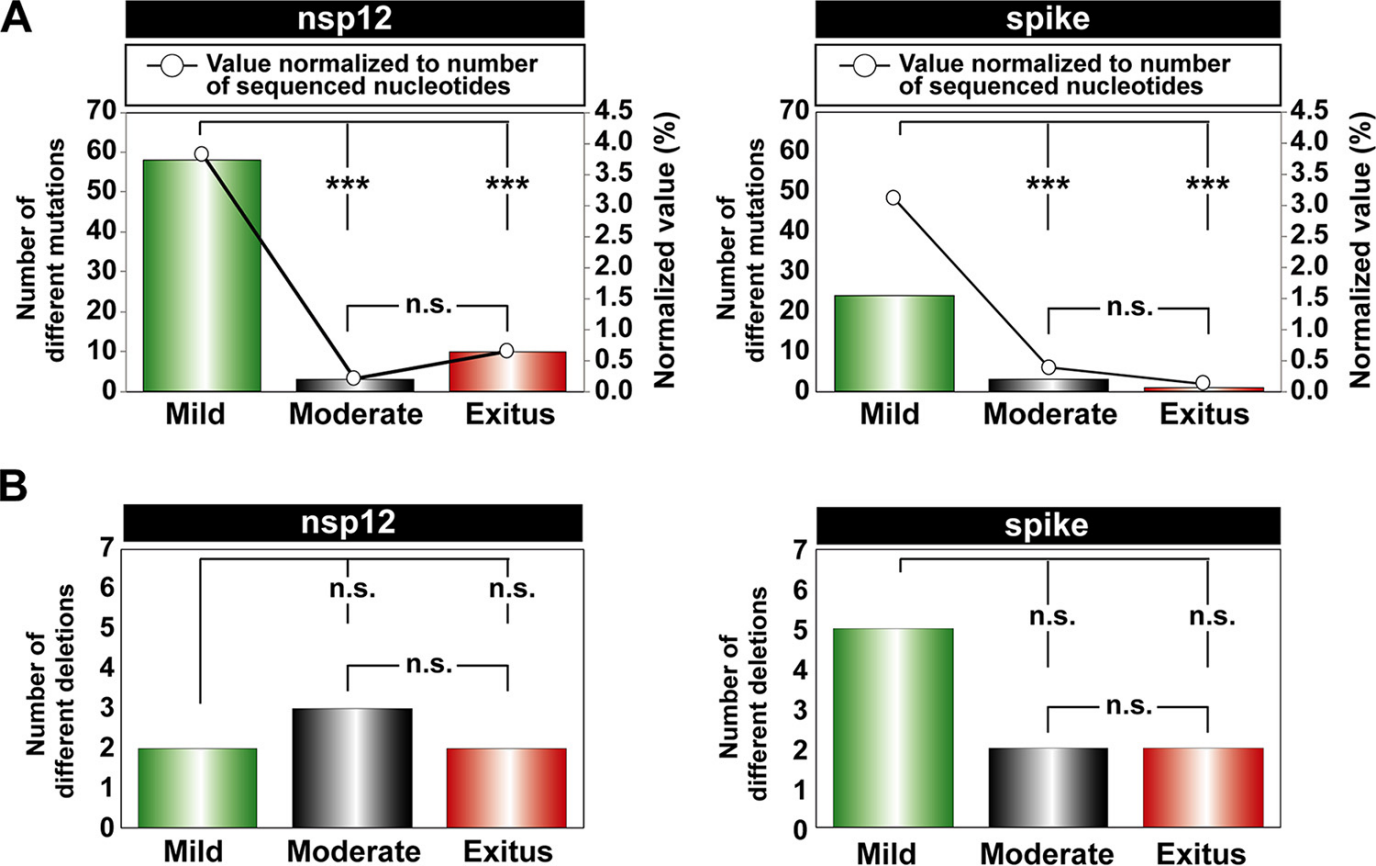
Point mutations and deletions in the mutant spectra of SARS-CoV-2 isolates, distributed according to COVID-19 severity. The point mutations and deletions are those depicted in Fig. 2. (A) Total number of different point mutations in the nsp12 (polymerase)-coding (left panel) and the S-coding (right panel) regions distributed according to disease severity (mild, moderate, exitus, as indicated in the abscissa) in the patients from whom the virus was isolated. Bars indicate the total absolute number of mutations (left ordinate axes), and empty dots give the percentages normalized to the length in nucleotides of the sequenced regions (right ordinate axes). (B) Total number of different deletions in the nsp12 (polymerase)-coding (left panel) and the S-coding (right panel) regions distributed according to disease severity in the patients from whom the virus was isolated. For panels A and B the statistical significance of the differences was determined by the proportion test; ns; not significant; ***, *P* < 0.001.

To evaluate if some parameters of the mutant spectra (considering only point mutations present at a frequency below 30%) were associated with COVID-19 severity, we first counted the number of different point mutations present in virus from each patient group. In the two coding regions analyzed, the average number of different mutations in virus from patients with mild disease was significantly higher than that in virus from patients with moderate disease or exitus (*P* < 0.001 for the comparison between mild versus moderate and mild versus exitus, both for nsp12 [polymerase]- and S-coding regions; proportion test). No significant difference was noted between moderate and exitus patients (*P* = 0.081 and *P* = 0.603 for nsp12 [polymerase]- and S-coding regions, respectively; proportion test); normalization of the number of different mutations to the length of the regions analyzed did not modify the result (Fig. 3A). No such difference among patient groups was observed with the number of different deletions (all *P* values were >0.05; proportion test), although a trend toward a larger number of deletions in virus from patients who developed mild disease was maintained in the S-coding region (Fig. 3B). Thus, SARS-CoV-2 mutant spectra from diagnostic samples of patients who evolved to mild disease included a significantly larger average number of mutations, but not of deletions, than virus from patients who progressed toward moderate or severe (exitus) COVID-19.

### Evaluation of complexity indices.

**FIG 4:**
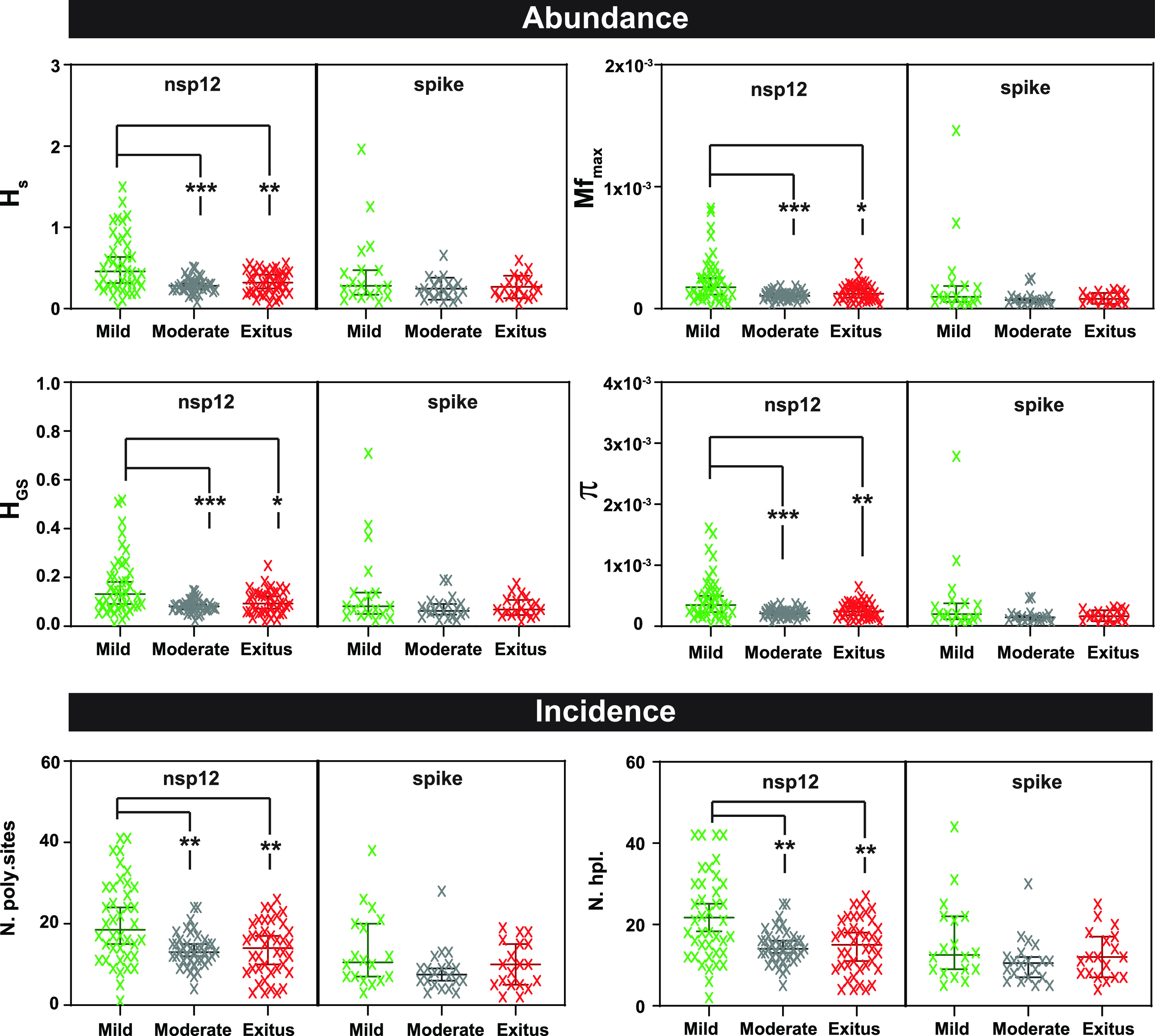
Comparison of the diversity indices for all amplicons of either the nsp12 (polymerase)- or S-coding region, distributed according to virus-associated disease severity. The types of indices (abundance or incidence) are indicated in the heading filled boxes. The specific index is indicated in ordinate (13) (H_s_, Shannon entropy; Mf_max_, maximum mutation frequency; H_GS_, Gini Simpson; π, nucleotide diversity; N. poly.sites, number of polymorphic sites; N. hpl., number of haplotypes). Each cross is the numerical value obtained for the virus of an individual patient; patients have been distributed according to disease severity as indicated in abscissa (color coded). Data were obtained using a cutoff value of 0.1%, as reported previously (13). Values for each amplicon and patient are compiled in Table S3 in https://saco.csic.es/index.php/s/8GH5aJgritCjEx5. The statistical significance of the differences has been determined by the Wilcoxon test. *, *P < *0.05; **, *P < *0.01; ***, *P < *0.001; absence of connecting lines means that the difference between two patient groups was not statistically significant.

The comparison of SARS-CoV-2 mutant spectra was extended to two groups of diversity indices: abundance (which consider the reads of entities and their frequency in the mutant spectrum) and incidence (which consider only reads of entities) (13). To this aim, we have adapted the QSutils package (43) to the quantification of diversity indices for SARS-CoV-2 mutant spectra (described in Materials and Methods). In the nsp12 (polymerase)-coding region, a significant increase of the values of abundance and incidence indices was observed in samples from patients who developed mild disease, compared with those of samples from patients with moderate disease (*P < *0.001 for H_s_ [Shannon entropy], H_GS_ [Gini Simpson], Mf_max_ [maximum mutation frequency], and π [nucleotide diversity]; *P = *0.001 for number of polymorphic sites and number of haplotypes; Wilcoxon test). Also significant was the difference between samples associated with mild disease and severe disease (exitus) (*P = *0.004 for H_S_, *P = *0.010 for H_GS_, *P = *0.012 for Mf_max_ and *P = *0.010 for π; *P = *0.004 for number of polymorphic sites and number of haplotypes; Wilcoxon test). The same tendency was observed in the S-coding region, but the differences did not reach statistical significance (all *P* values were >0.05; proportion test) (Fig. 4 and Table S3 in https://saco.csic.es/index.php/s/8GH5aJgritCjEx5). In each amplicon, a larger number of haplotypes was found in samples associated with mild disease than in those associated with moderate or severe disease, and the majority of mutated haplotypes included only one mutation (Fig. S2 in https://saco.csic.es/index.php/s/8GH5aJgritCjEx5). Thus, the higher abundance of mutations in SARS-CoV-2 mutant spectra from patients who exhibited only mild symptoms is also reflected in an increase of mutant spectrum complexity.

### Point mutation and amino acid substitution types in SARS-CoV-2 mutant spectra.

Considering mutant spectra of all samples analyzed, transitions and nonsynonymous mutations were more abundant than transversions and synonymous mutations, respectively, with different degrees of statistical significance (Table 1); a similar trend was also observed when the samples were divided according to COVID-19 severity of the patients.

**TABLE 1:** Point mutations in the mutant spectra of SARS-CoV-2 isolates^*a*^

Characteristics	Result for patients with disease severity			
	**Total**	**Mild**	**Moderate**	**Exitus**
nsp12				
Transitions (%)	68 (97.14%)	56 (96.55%)	3 (100%)	10 (100%)
Transversions (%)	2 (2.86%)	2 (3.45%)	0 (0%)	0 (0%)
*P* value	<0.001	<0.001	0.051	<0.001
Significance^*b*^	***	***	n.s.	***
Synonymous (%)	29 (41.43%)	24 (41.38%)	2 (66.67%)	4 (40%)
Nonsynonymous (%)	41 (58.57%)	34 (58.62%)	1 (33.33%)	6 (60%)
*P* value	0.031	0.047	0.5	0.327
Significance^*b*^	*	*	n.s.	n.s.
Spike				
Transitions (%)	26 (96.30%)	24 (100%)	3 (100%)	0 (0%)
Transversions (%)	1 (3.70%)	0 (0%)	0 (0%)	1 (100%)
*P* value	<0.001	<0.001	0.051	0.5
Significance^*b*^	***	***	n.s.	n.s.
Synonymous (%)	11 (40.74%)	10 (41.67%)	1 (33.33%)	0 (0%)
Nonsynonymous (%)	16 (59.26%)	14 (58.33%)	2 (66.67%)	1 (100%)
*P* value	0.138	0.193	0.5	0.051
Significance^*b*^	n.s.	n.s.	n.s.	n.s.

a Different number of point mutations distributed according to COVID-19 severity in the nsp12 (polymerase)- and spike-coding regions.

b The statistical significance of the differences (n.s., not significant; *, *P* < 0.05; ***, *P* < 0.001) was calculated using the proportion test.

In the nsp12 (polymerase)-coding region, the frequency of mutation types normalized to base composition ranked as follows: T to C > A to G > C to T. When dividing the samples according to disease severity, the most frequent mutation in exitus patients was C to T (Fig. S3A in https://saco.csic.es/index.php/s/8GH5aJgritCjEx5). In the S-coding region, the ranking was T to C > A to G = C to T (Fig. S3B in https://saco.csic.es/index.php/s/8GH5aJgritCjEx5). T to C transitions were the most frequent mutation type in the third codon position (67.50%), whereas A to G was the most prevalent type at the second and first codon positions (45.16% and 38.46%, respectively).

**FIG 5:**
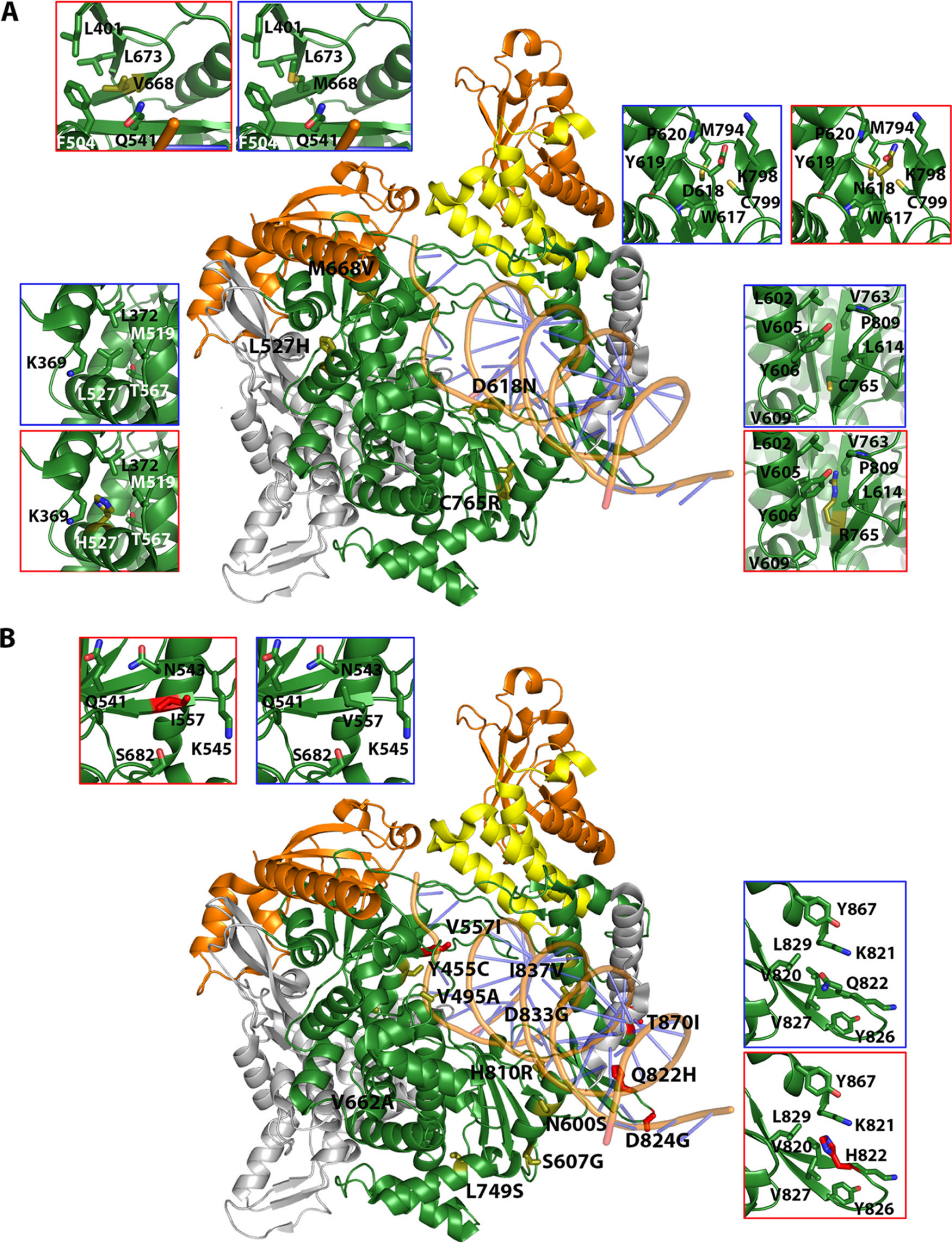
Location of amino acid substitutions in the three-dimensional structure of nsp12 (polymerase). The structure used as reference is that of the replication complex nsp12-nsp8-nsp7 (PDB code 6NUR with the RNA superimposed from 7CYQ). (A) Substitutions found at low frequency (0.5% to 2%) in the mutant spectra. The central structure is a cartoon representation of the nsp12, depicted in gray and green, the latter showing the regions covered by amplicons A1 to A4 (indicated in Fig. 1). Contact proteins nsp8 (orange) and nsp7 (yellow) are also drawn. Substitutions are labeled, and amino acids are shown as sticks in different colors, according to associated disease category: exitus in red, mild disease in yellow. Insets highlight the interactions of some substitutions with neighboring residues within a 5-Å radius. Two insets are shown per position, indicating the original and mutated residues, squared in blue and red, respectively. (B) Same design as in panel A but with substitutions found at frequency higher than 2%. The substitutions and their frequency in the mutant spectrum, acceptability, functional score, and possible structural or functional effects are listed in Table 2.

**FIG 6:**
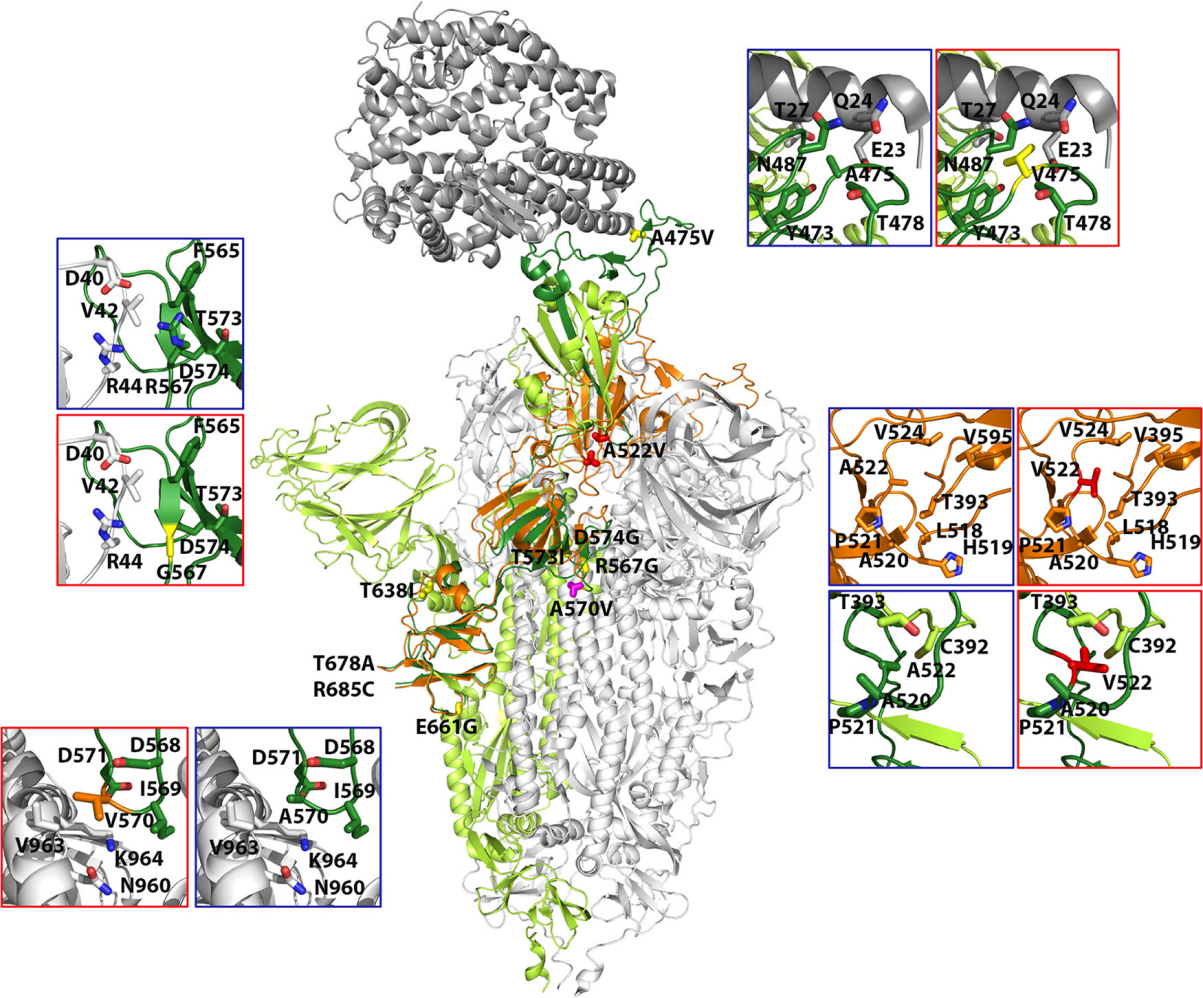
Location of amino acid substitutions in the three-dimensional structure of spike (S) protein. The central structure is a cartoon representation of S trimer (PDB code 7A94) with the reference monomer colored in green and dark green, the latter marking the regions covered by amplicons A5 to A6 (indicated in Fig. 1). The remaining monomers of the S trimer are shown in gray. The reference monomer contains the RBD domain in the “erect” position. A superimposition of this domain in the “open” conformation is also shown in orange. Substitutions are labeled, and amino acids are shown as sticks in different colors, according to associated disease category: exitus in red; moderate in magenta, and mild in yellow. Insets highlight the interactions of some substituted positions with neighboring residues within a 5-Å radius. Except for position 522, two insets are shown per mutated position, indicating the original and mutated residues, squared in blue and red, respectively. For position 522, four insets are shown; the top two indicate the interactions of this residue in the open conformation of RBD, and the bottom two indicate those in the erect conformation. The substitutions and their frequency in the mutant spectrum, acceptability, functional score, and possible structural or functional effects are listed in Table 3.

The amino acid substitutions found in nsp12 (polymerase) and S were positioned in the three-dimensional structure of the proteins (Protein Data Bank [http://www.wwpdb.org/]), their statistical acceptability was evaluated with PAM250 matrix (44), and their potential functional effects were estimated by applying the SNAP2 predictor (45). All amino acid substitutions found in nsp12 (polymerase) and S are listed in Table S2 in https://saco.csic.es/index.php/s/8GH5aJgritCjEx5, together with their PAM250 and SNAP2 scores; their location in the three-dimensional structure of the proteins is depicted in Fig. S4 in https://saco.csic.es/index.php/s/8GH5aJgritCjEx5. Those amino acid substitutions which suggest alteration of protein structure or function are described in Tables 2 and 3. Some of the substitutions in nsp12 (polymerase) predict positive or negative functional effects (Table 2 and Fig. 5). For example, V557I may enhance the stability of the interaction with nitrogen base T + 1, and Q822H predicts increased stability of loop in the thumb domain. In contrast, D618N abolishes the catalytic aspartate of polymerase in domain A, and C765R should distort the catalytic domain (Table 2 and Fig. 5). The amino acid substitutions observed in S tend to increase the hydrophobicity of the region where they are located (Table 3 and Fig. 6). The replacement of A by V at position 475 may enhance interactions of S with ACE2, A522V may contribute to stabilize the RBD domain in the “open” position through contacts with neighbor V, T, P, and L residues, R567G could facilitate fusion with the host cell, and A570V may bring closer two S chains (Table 3 and Fig. 6). Drastic substitutions may belong to defective genomes that have a transient existence or that may be maintained by complementation (see Discussion).

**TABLE 2:** Amino acid substitutions at the nsp12 (polymerase) in the mutant spectra of SARS-CoV-2^*a*^

Substitution and patient category	Patient category	PAM250	SNAP2 (score)	Location and possible structural or functional effect(s)
Low-frequency substitutions (0.5%–2%)				
V373A	Mild	0	Neutral (−55)	Interface, between NiRan and fingers. Loss of a side chain that may interact with L527 and I536 (at 4 Å and 3.8 Å, respectively) of fingers domain.
D499G	Mild	1	Effect (70)	RNA template binding region, but not in direct contact with RNA. May enhance RNA binding through increase in electropositivity.
L514P	Mild	−3	Neutral (−34)	Near V83 of nsp7. Could affect the interaction between nsp7 and nsp12 polymerase, although other nsp12 residues (F368, L372, F506) are also involved.
L527H	Mild	−2	Effect (51)	Fingers’ helix in contact with the NiRan. It may require structural accommodation in a hydrophobic environment.
V560A	Mild	0	Effect (3)	Palm, motif B side chain of V560 interacts with S681, generating the up/down positioning of loop B, involved in RNA translocation, as described in picornaviruses (91). The V-A substitution would inhibit this interaction.
D618N	Mild	2	Effect (75)	Catalytic D of motif A. Loss of polymerization function.
N628S	Mild	1	Neutral (−35)	Fingers. Establishes links with a helix and a loop from fingers through salt bridges. S628 breaks the links and may increase domain flexibility.
M668V	Mild	2	Neutral (−54)	Exposed residue in the template entry channel. Substitution M-V would lead to an expansion of the channel.
L727P	Mild	−3	Effect (66)	Lower part of palm domain. A P residue in this position fits well into a region rich in aromatic amino acids.
C765R	Mild	−4	Effect (76)	β-Strand of the hairpin forming motif A that includes the active site. R at this position would disrupt the surroundings of the active site, probably inducing a nonfunctional protein.
L372F	Exitus	2	Effect (8)	Interface, between NiRan and fingers. F may reinforce the hydrophobic environment.
Medium-frequency substitution (2%–30%)				
V557I	Exitus	4	Neutral (−51)	Close to the entry of the RNA template channel. In contact with the nitrogen base T + 1. An I may enhance the stability of this connection.
High-frequency substitution (>90%)				
Q822H	Exitus	3	Neutral (−85)	In a loop of the thumb domain. An H could enhance loop stability.

a The sequenced region spans amino acids 366 to 871. Substitutions are divided according to the frequency at which they are found in the mutant spectra and disease category (mild, moderate, or severe [exitus] as defined in Materials and Methods) (Fig. 2). PAM250 and SNAP2 scores have been calculated as described in references 44 and 45, respectively. Possible structural effects have been predicted from the location of the substitution in the three-dimensional structure of nsp12 (polymerase) (Fig. 5).

**TABLE 3:** Amino acid substitutions at the spike (S) protein in the mutant spectra of SARS-CoV-2^*a*^

Substitution	Patient category	PAM250	SNAP2 (score)	Location and possible structural or functional effect(s)
Low-frequency substitutions (0.5%–2%)				
R567G	Mild	−3	Effect (47)	Contact region, involved in the formation of the S trimers. This substitution would eliminate the R567-D40 salt bridge, involved in regulation of the viral fusion. The R-G substitution could facilitate fusion with cell.
T573I	Mild	0	Neutral (−54)	β-chain next to R567 and close to a V and two F residues. The T-I substitution may strengthen hydrophobic contacts in the region.
D574G	Mild	1	Neutral (−39)	β-Chain next to T573. Loss of contact with K557 and increase of flexibility.
E661G	Mild and moderate	0	Effect (41)	Exposed residue that could interact with Q779 of another chain in the S trimer. A G would prevent this interaction.
Medium-frequency substitutions (2%–30%)				
A475V	Mild	0	Neutral (−88)	Interaction with ACE2. V may increase contact with ACE2 receptor.
T678A	Mild	1	Neutral (−23)	Loop near the furin cleavage site. Expected to be exposed upon furin cleavage. However, this region appears disordered in the deposited structures.
R685C	Mild	−4	Effect (26)	Furin cleavage site (PRRAR). A C would either inhibit the cleavage or decrease the efficacy of the excision, thus hindering the S1/S2 excision.
A570V	Moderate	0	Neutral (−88)	Interaction region to form S trimers. V could bring closer the two chains due to its larger and more hydrophobic side chain.
High-frequency substitution (>90%)				
A522V	Exitus	0	Neutral (−71)	Loop close to the hinge, linking the RBD and the subdomain 1 of S1. This loop facilitates the transition from the “open” to the “erect” position of the RBD. The A-V substitution may enhance the stability of the RBD open position, due to its proximity to other hydrophobic residues.

a The sequenced region spans amino acids 438 to 694. Substitutions are divided according to the frequency at which they are found in the mutant spectra and disease category (mild, moderate, or severe [exitus] as defined in Materials and Methods) (Fig. 2). PAM250 and SNAP2 scores have been calculated as described in references 44 and 45, respectively. Possible structural effects have been predicted from the location of the substitution in the three-dimensional structure of S (Fig. 6).

### Deletion repertoire in SARS-CoV-2 mutant spectra.

Deletions were also analyzed by UDS with a cutoff value of 0.5% (as detailed in Materials and Methods), with the same reads used for point mutations. The analyses identified five different deletions which spanned 3 to 13 nucleotides (nt) in the nsp12 (polymerase)-coding region and five different deletions that spanned 2 to 51 nt in the S-coding region (Fig. 2 and S5 in https://saco.csic.es/index.php/s/8GH5aJgritCjEx5). In the nsp12 (polymerase)-coding region, the 4-nt and 13-nt deletions that disrupted the coding frame generated a stop codon 10 and 26 residues downstream, respectively. The 2-nt, 16-nt, 22-nt, and 28-nt deletions in the S-coding region led to stop codons 3 to 18 nucleotides downstream (Fig. S5 in https://saco.csic.es/index.php/s/8GH5aJgritCjEx5). The number of deletions that generated a stop codon was significantly higher in the S-coding region (26 out of 27 deletions) than in the nsp12 (polymerase)-coding region (2 out of 10 deletions) (*P < *0.001; proportion test). The sites of deletions did not map in homopolymeric regions or tandem repeats, and they were not flanked by the same nucleotide types (Fig. S5 in https://saco.csic.es/index.php/s/8GH5aJgritCjEx5).

### Point mutation and deletion hot spots.

**FIG 7:**
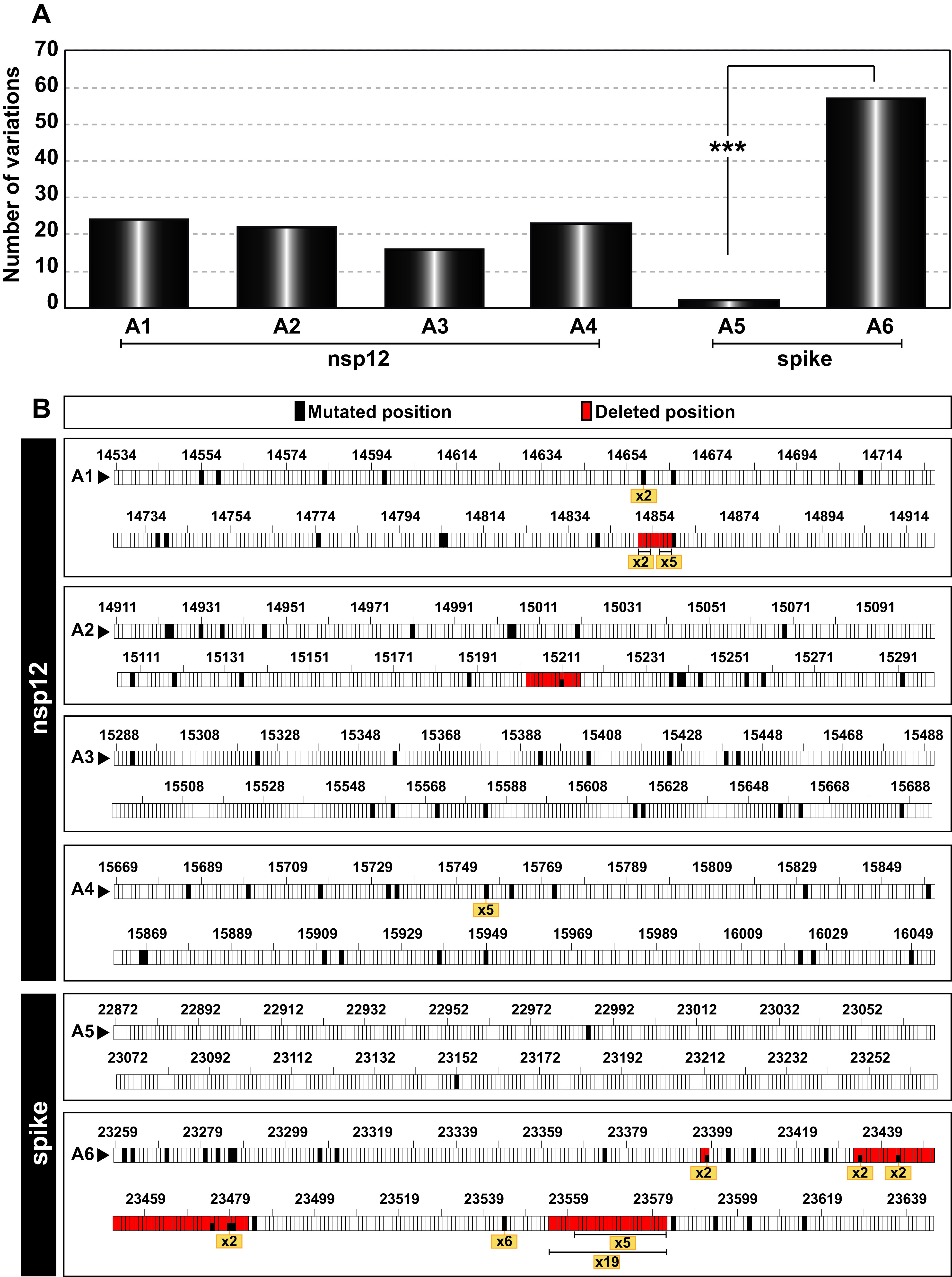
Point mutation and deletion hot spots in SARS-CoV-2 mutant spectra. (A) Distribution of the total number of different variations (point mutations and deletions, given in ordinate) among the amplicons analyzed (indicated in abscissa). The statistical significance of the differences was determined by the proportion test. ***, *P < *0.001; absence of connecting lines among nsp12 amplicons means that differences were not statistically significant. (B) Location of point mutations and deletions within each amplicon (indicated in each box). Genome residue numbering is according to reference NCBI accession number NC_045512.2. The numbers written in a yellow box refer to the number of patients whose virus carried the same mutation or deletion and serve to identify hot spots. Point mutations and deletions were counted relative to the consensus sequence of the corresponding population.

The distribution of genomic variations (point mutations and deletions) per amplicon was similar for the four amplicons of the nsp12 (polymerase)-coding region (*P* value > 0.05; proportion test). In contrast, amplicon A6 of the S-coding region accumulated a higher number of total mutations than A5 (*P < *0.001; proportion test) (Fig. 7A). This difference may result from dissimilar functional constraints on the protein portions represented by each amplicon, i.e., a uniform distribution of polymerase motifs A to G among the four nsp12 (polymerase) amplicons, compared with the presence of the receptor-binding domain (RBD) in amplicon A5 of S (compare Fig. 1 and Fig. 7A).

Hot spots for SARS-CoV-2 variations have been described based on the comparison of consensus sequences of independent isolates (46–48). Here, we have defined as hot spots those positions that presented the same point mutation or deletion in the mutant spectrum of at least five different isolates (Fig. 7B and Table S2 in https://saco.csic.es/index.php/s/8GH5aJgritCjEx5). Two hot spots were located in the nsp12 (polymerase)-coding region (a point mutation at position 15,756 and a deletion of residues 14,856 to 14,858), and two were located in the S-coding region (a point mutation at position 23,544 and a deletion of residues 23,555 to 23,582) (Fig. 7B). These hot spots do not coincide with those reported for SARS-CoV-2 consensus sequences (46–48).

### Geographical and temporal characterization of mutations based on CoV-GLUE database.

SARS-CoV-2 mutant spectra from infected patients can include mutations that are also found as dominant in later isolates (27). In the mutant spectra of the 30 samples from our cohort, the ratio of amino acid substitutions (including those corresponding to divergence mutations) that were unique (not yet annotated in the CoV-GLUE database that is enabled by GISAID metadata [49]) versus those described in other (prior or subsequent) isolates was 0.2 (10 out of 60). Out of the 60 nonsynonymous mutations, 8 (13.33%) were described worldwide at about the same time that they were identified in our cohort, and 19 (31.67%) were described afterwards (Fig. S6 in https://saco.csic.es/index.php/s/8GH5aJgritCjEx5). Of particular interest is S protein substitution S494P, which is located at the ACE-2 binding region (Table S2 in https://saco.csic.es/index.php/s/8GH5aJgritCjEx5) that reached epidemiological importance and was found in some isolates of the alpha variant. Thus, SARS-CoV-2 mutant spectra—in particular those from patients that developed mild symptoms—may constitute a rich reservoir of mutations with the potential to be represented in epidemiologically relevant variants.

## DISCUSSION

The UDS analysis of the nsp12 (polymerase)- and S-coding regions of 30 biological samples without cell culture passage confirmed the presence of complex SARS-CoV-2 mutant spectra in diagnostic nasopharyngeal samples of the virus (23–28). Contrary to a previous conclusion with other patient cohorts (35, 36), our quantifications show that in both the nsp12 (polymerase)- and the S-coding regions analyzed, there was a positive association between the number of point mutations and a mild disease manifestation in the corresponding patients. No such association was observed with the minority deletions that also populated the mutant spectra (Fig. 2 and 3). There are several non-mutually exclusive mechanisms that may contribute to a larger average number of point mutations in samples from patients that developed mild disease than in those from patients with moderate or severe disease. One is that the major sites of replication of the virus may not be identical in the three groups of patients. Mutational input may be affected by a variety of host cell functions, including editing activities (50), or as a consequence of the effects on polymerase fidelity of nonstructural viral proteins that participate in genome replication, as evidenced with other RNA viruses (51–54). This possibility for SARS-CoV-2 is suggested by nonidentical preferred transition mutation types in the isolates, depending on the associated disease severity (Table 1). A second influence may lie in a longer time of asymptomatic intrahost virus replication prior to the onset of mild symptoms and COVID-19 diagnosis. A prolonged replication time does not necessarily imply a larger viral load in the infected host, but it may entail an increase in the average number of variant genomes in the population. Another possibility is that bottleneck events, which may transiently reduce the number of mutations scored within mutant spectra, intervene with higher intensity in patients doomed to severe disease than in those developing mild disease. This may come about through the immune response that may partially suppress viral replication and that it is also part of the COVID-19 pathogenesis process (55–57). Several possibilities may explain dissimilar conclusions with other studies; for example, (i) independent cohorts may have been infected by virus belonging to clades displaying nonidentical behavior, and (ii) there may have been methodological differences, such as in the criteria to classify patients according to COVID-19 symptoms, in the PCR-UDS resolution attained, or in the sample type taken for analysis (naso/oropharyngeal swabs versus nasopharyngeal aspirates), among others. The multiple factors that contribute to a mutant spectrum complexity beg for studies with other cohorts to try to clarify whether complexity of viral RNA in diagnostic samples responds to discernible virological parameters and whether UDS data might help predict disease evolution or response to treatment, as previously documented for hepatitis C (58, 59).

We have focused the mutant spectrum analysis on two regions of the SARS-CoV-2 genome whose encoded proteins are likely subjected to widely different constraints. The nsp12 (polymerase) is involved in genome replication and transcription, and the S glycoprotein has a major role in virus attachment, fusion, and entry, as well as in defining the antigenic profile of the virus. A total of 41 different amino acid substitutions in nsp12 and 15 substitutions in S have been recorded in the 30 mutant spectra analyzed (Table S2 in https://saco.csic.es/index.php/s/8GH5aJgritCjEx5). Normalization to the sequenced protein length gives an average frequency of nonsynonymous mutations of 8% for nsp12 and 6% for S in the mutant spectra. Three substitutions in S map in the receptor-binding domain (RBD). One of them, A475V (present at 26% frequency in virus from a patient who developed mild disease), reduced the sensitivity to several monoclonal antibodies (60). S494P (dominant in virus from a patient who developed mild disease) was listed among the nine most frequent substitutions in a large-scale study of 506,768 SARS-CoV-2 isolates; it is considered a likely vaccine-escape substitution and is possibly also involved in increased transmissibility of some isolates of the alpha variant detected beginning September 2020 (61–63) (https://www.cdc.gov/).

Substitutions that are present at low frequency are predicted to have more drastic structural and functional effects, and some of them have been identified in the sequences compiled in the CoV-GLUE database (compare Table 2 and Fig. S6 in https://saco.csic.es/index.php/s/8GH5aJgritCjEx5).

It is likely that disruptive amino acid substitutions belong to defective or minimally replicating (very low fitness) genomes that either have a transient existence in the population or can be maintained at detectable levels by complementation (for example those with lesions incompatible with polymerization activity) (64). Defective genomes need not represent a biological or evolutionary dead end. They can exert modulatory effects on the entire population (65), and they also constitute a rich substrate for RNA recombination to rescue viable genomes that may become epidemiologically competent viruses.

Newly replicated genomes *in vivo* may incorporate deletions as a result of limited processivity of the coronavirus replicase (66, 67). Genomes with deletions may, on average, be subjected to stronger negative selection than genomes with point mutations, blurring differences in their frequency among samples from the three patient categories. This is likely to apply mainly to out-of-frame deletions that give rise to truncated proteins; for example, in the S-coding region, we have identified deletions (Δ)23,555 to 23,570, Δ23,555 to 23,582, and Δ23,561 to 23,582, which are located near the S1/S2 cleavage site and are expected to impair S function. Their maintenance to the point of reaching sufficient concentration to be detectable by UDS may reflect an efficiency of complementation of *trans*-acting structural proteins higher than that of nonstructural proteins (64). This may also explain the lower frequency of out-of-frame deletions in the nsp12 (polymerase)- than in the S-coding region. It has been proposed that defective S proteins generated around the S1/S2 cleavage site could potentially reduce the severity of the infection (68).

All point mutations and deletions were found at frequencies below 30% in the corresponding mutant spectra. Several important biological and clinical features could influence the shape of SARS-CoV-2 mutant spectra. However, it should be considered that the large size of the coronavirus genome may limit the accumulation of mutations relative to that of less complex RNA genomes due to negative effects of mutations on fitness (69). Not even the point mutation hot spots were found at frequencies above 1% in the quasispecies where they were present (compare Fig. 2 and 7). This is compatible with hot spots reflecting sites where lesions are more tolerated within a generally constrained RNA genome. The fact that hot spots according to mutant spectra do not coincide with those defined by consensus sequences adds to other observations that indicate that residue conservation criteria at these two levels do not coincide (70). The fact that the great majority of mutations in SARS-CoV-2 mutant spectra are present at low frequency may slow down the response of the virus to specific selective constraints such as inhibitors or neutralizing antibodies. Under this scenario, viral load may become more important to furnish genomes with mutations required to respond to the constraints (71). Comparative measurements with different RNA viruses are needed to endorse these potential effects of mutant spectrum composition.

The higher percentage of transitions versus transversions, and of nonsynonymous versus synonymous mutations, is in agreement with previous reports of mutant spectrum and consensus sequence analyses of SARS-CoV-2 (23, 24, 35, 68, 72). Some differences with previous studies have been observed in the preferred mutation types (Fig. S3 in https://saco.csic.es/index.php/s/8GH5aJgritCjEx5). While in the mutant spectra of our cohort T to C was the most frequent point mutation, other studies reported C to T as the preferred mutation type (72, 73). C to T was, however, the most frequent mutation in virus from the subset of exitus patients (Fig. S3 in https://saco.csic.es/index.php/s/8GH5aJgritCjEx5), hinting at the possibility that in previous studies virus from patients with moderate and severe COVID-19 might have been overrepresented. The lack of dominance of C to U transitions in our samples is also reflected in the absence of depletion of amino acids A, H, Q, P, and T when considering all amino acid substitutions observed (50, 74); the data of Fig. S3 in https://saco.csic.es/index.php/s/8GH5aJgritCjEx5 show a net gain of 3 amino acids in the A, H, Q, P, and T subset. Another possible explanation for differences with previous studies could be that the previous studies focused on consensus sequences obtained from data bases covering the whole genome, whereas our results correspond to two specific genomic regions sequenced by UDS.

The six point mutations that altered the consensus sequence of the mutant spectra relative to that of the reference, NC_045512.2 (identified as “Divergence” in the heat map of Fig. 2 and in Table S2 in https://saco.csic.es/index.php/s/8GH5aJgritCjEx5), allowed an estimate of the rate of accumulation of mutations in the SARS-CoV-2 consensus sequence. The time interval between our Madrid isolates (dated April 2020) and the reference Wuhan isolate (dated December 2019) was 4 months. Considering this time interval, the average rate of evolution calculated is (1.6 ± 0.6) × 10^−3^ mutations per nucleotide and year (m/n/y), and it is only slightly higher than the average value from 10 previous studies: (1.2 ± 0.6) × 10^−3^ m/n/y (range 9.9 × 10^−4^ to 2.2 × 10^−3^ m/n/y) (73, 75–83). Higher evolutionary rates are frequently obtained as the time intervals between the virus isolations considered for the calculation become shorter (reviewed in reference 84). The values for SARS-CoV-2 are comparable to those reported for other RNA viruses, suggesting that constraints at the quasispecies level may not affect significantly evolutionary rates considered at the epidemiological level (85). Our results hint at the possibility that SARS-CoV-2 evolving in patients exhibiting mild symptoms may contribute a majority of the variants that drive the high rates of evolution quantified at the epidemiological level.

## MATERIALS AND METHODS

### Patient cohort and stratification.

Samples were collected during the first COVID-19 outbreak in Spain. The cohort of the study included 30 patients admitted to the Fundación Jiménez Díaz Hospital (FJD, Madrid, Spain) from 3 to 29 April 2020. All patients were confirmed to be positive for SARS-CoV-2 by a specific real-time PCR (VIASURE) with a *C_T_* (cycle threshold, which is inversely correlated with viral RNA level) range of 15.6 to 28.5; the samples are a subset from the cohort that has been described previously in reference 37. Data collected included patient demographics, risk factors for COVID-19, and clinical information at the time of SARS-CoV-2 diagnosis (Table S1 in https://saco.csic.es/index.php/s/8GH5aJgritCjEx5). The parameters used to classify the patients included (i) need of hospitalization, (ii) need of mechanical ventilation, (iii) admission to the intensive care unit (ICU), and (iv) exitus attributed to COVID-19. Taking these parameters into account, the patients were classified as mild, moderate, and severe (exitus) cases according to the symptoms and hospitalization requirements: (i) mild symptoms (neither hospital admission nor ICU) (*n *= 10), (ii) moderate symptoms (hospitalization without ICU) (*n *= 10), and (iii) severe symptoms (hospitalization with admission to the ICU and progression to exitus in all cases) (*n *= 10). The clinical classification was established before the data analysis was performed.

### Oligonucleotide design.

To design oligonucleotide primers, we retrieved a total of 663 SARS-CoV-2 sequences from the NCBI database (https://www.ncbi.nlm.nih.gov/genbank/sars-cov-2-seqs/) and aligned them to the Wuhan-Hu-1 NCBI reference sequence NC_045512.2 (86). Nucleotide sequences were analyzed to design forward and reverse oligonucleotide primers (Table S4 in https://saco.csic.es/index.php/s/8GH5aJgritCjEx5). Four pairs of oligonucleotides were used for amplification and sequencing of four overlapping amplicons of the genomic region of nsp12 (polymerase) (nucleotides 14,511 to 16,075) encoding amino acids 366 to 871, and two pairs were used to cover the region of the S protein (nucleotides 22,853 to 23,666) encoding amino acids 438 to 694 (residue numbering according to reference sequence NC_045512.2) (Fig. 1 and Table S5 in https://saco.csic.es/index.php/s/8GH5aJgritCjEx5).

### RNA extraction and amplification of SARS-CoV-2 RNA from infected patients.

SARS-CoV-2 RNA was extracted from 140 μL of medium from nasopharyngeal swabs using the QIAamp Viral RNA minikit (250) (Qiagen), as specified by the manufacturer. Amplifications of nsp12 (polymerase)- and S-coding regions were performed by reverse transcriptase (RT)-PCR. Each region was amplified from 5 μL of the RNA preparation by RT-PCR using Transcriptor one step RT-PCR kit (Roche Applied Science). To perform the RT-PCR, 5 μL of the preparation was mixed with 10 μL of 5× buffer, 2 μL of a solution containing the forward primer, 2 μL of a solution with the reverse primer (50 ng/μL, each), and 1 μL of polymerase. Reaction parameters were 50°C for 30 min for the reverse transcription, an initial denaturing step at 94°C for 7 min, followed by 35 cycles of a denaturing step at 94°C for 10 s, an annealing step at 46 to 48°C for 30 s, an extension step at 68°C for 40 s, and then a final extension at 68°C for 7 min. In the case of samples with a *C_T_* value greater than 26 (6 samples from the mild symptom group), the number of cycles was increased to 45. Negative controls (amplification reactions in the absence of RNA) were included in parallel to ascertain absence of contamination by template nucleic acids. Amplification products were analyzed by 2% agarose gel electrophoresis, using GeneRuler 1 kb plus DNA ladder (Thermo Scientific) as molar mass standard. PCR products were purified (QIAquick gel extraction kit, Qiagen), quantified (Qubit dsDNA assay kit, Thermofisher Scientific), and tested for quality (TapeStation system, Agilent Technologies) prior to sequencing using the Illumina MiSeq platform. Dilutions of 1:10, 1:100, and 1:1,000 of the initial RNA preparation and subsequent amplification by RT-PCR were carried out for one patient of each disease severity (Fig. S7 in https://saco.csic.es/index.php/s/8GH5aJgritCjEx5). When amplification with the 1:1,000 dilution of template produced a visible DNA band, the ultradeep sequencing analysis was performed with the undiluted template to avoid redundant copying of the same template molecules, as we have documented previously (87, 88).

### Ultradeep sequencing of SARS-CoV-2 from infected patients.

We adjusted PCR products to 4 × 10^9^ molecules/μL before generating DNA pools that were purified using Kapa pure beads (Kapabiosystems, Roche), quantified them using Qubit as described previously (38–40), and then fixed them at 1.5 ng/μL. Purified DNA pools were further processed using the DNA library preparation kit Kapa hyper prep kit (Roche), during which each pool was indexed using SeqCap adapter kit A/B (Nimblegen; 24 Index). Each DNA pool was quantified by LightCycler 480 and sequenced using MiSeq sequencing platform with MiSeq reagent kit v3 (2 × 300 bp mode with the 600 cycle kit) (Illumina).

### Bioinformatics analyses.

Controls to establish the basal error, the frequency of PCR-induced recombination, and the similarity of the results with different amplifications and sequencing runs were performed previously (38, 41, 89). Therefore, mutations identified with a frequency above the 0.5% cutoff value and with coverage greater than 10,000 reads were considered for the analyses, based on different controls carried out with hepatitis C virus (HCV), as detailed elsewhere (38, 90).

Beginning with the Fastq data, two bioinformatic pipelines (SeekDeep [42] and a new previously described pipeline for HCV [38]) were applied to HCV (Fig. S8 in https://saco.csic.es/index.php/s/8GH5aJgritCjEx5) and then adapted to SARS-CoV-2 to quantify deletions (termed VQS-Haplotyper, freely available in Github at https://github.com/biotechvana/VQS-haplotyper) (Fig. S9 in https://saco.csic.es/index.php/s/8GH5aJgritCjEx5). As the control with an independent set of UDS data, we compared the point mutations and their frequencies within HCV quasispecies obtained using both bioinformatics procedures, and the results were very similar (*r *= 0.9957 and *P < *0.0001; Pearson correlation test) (Fig. S8 in https://saco.csic.es/index.php/s/8GH5aJgritCjEx5). For SARS-CoV-2 mutant spectra, the analysis of clean reads using both pipelines yielded a robust similar number of point mutations and their frequencies (*r *= 1 and *P < *0.0001; Pearson correlation test). Also, both pipelines produced similar results for deletions and their frequencies (*r *= 0.4932 and *P = *0.0011; Pearson correlation test) (Fig. S9 in https://saco.csic.es/index.php/s/8GH5aJgritCjEx5). SeekDeep was applied using the following options: “--extraExtractorCmds=--checkRevComplementForPrimers --primerNumOfMismatches 3” “--extraProcessClusterCmds=--fracCutOff 0.001 --rescueExcludedLowFreqHaplotypes --rescueExcludedOneOffLowFreqHaplotypes” (42). In the present study, point mutations, deletions, and their frequencies were reported using SeekDeep, and diversity indices were calculated using VQS-Haplotyper followed by QSutils (43).

### Statistics.

The correlation between results obtained by the bioinformatics pipelines was calculated using Pearson’s correlation. The statistical significance of difference between the number and type of mutations in mild, moderate, and exitus patients as well as the differences between type of nucleotide changes and between PAM250 (accepted point mutations 250) and SNAP2 (screening for nonacceptable polymorphisms 2) values for amino acid substitutions were calculated by the proportion test. Statistics were inferred using software R version 4.0.2. The normality of data was tested with the Shapiro-Wilk normality test, and the statistical significance of differences between diversity indices was calculated with a Wilcoxon test using GraphPad Prism 8.00.

### Ethics approval and consent to participate.

This study was approved by the Ethics Committee and the Institutional Review Board of the FJD hospital (no. PIC-087-20-FJD).

### Data availability.

The reference accession numbers of sequences retrieved from NCBI used to design oligonucleotide primers are given in Table S4 in https://saco.csic.es/index.php/s/8GH5aJgritCjEx5. Fastq files of SARS-CoV-2 samples included in the patient cohort are available in ENA under project ID PRJEB48766. Nucleotide and amino acid replacements in SARS-CoV-2 from infected patients have been compiled in Table S2 in https://saco.csic.es/index.php/s/8GH5aJgritCjEx5.
